# Medicinal Amazonian Oleoresins: An Eco-Friendly Chemical Fingerprinting Method

**DOI:** 10.3390/plants15111651

**Published:** 2026-05-28

**Authors:** Rayssa Ribeiro, Gabriel Reis Alves Carneiro, Henrique Marcelo Gualberto Pereira, Monica Costa Padilha, Valdir F. Veiga-Junior

**Affiliations:** 1 Military Institute of Engineering, Praça General Tibúrcio, 80, Praia Vermelha, Urca, Rio de Janeiro 22290-270, RJ, Brazil; rayssaribeiro_92@hotmail.com; 2 Brazilian Doping Control Laboratory (LBCD/IQ—UFRJ), Chemistry Institute, Federal University of Rio de Janeiro, Rio de Janeiro 21941-909, RJ, Brazil; gabriel.carneiro@iq.ufrj.br (G.R.A.C.); henriquemarcelo@iq.ufrj.br (H.M.G.P.); monicapadilha@iq.ufrj.br (M.C.P.)

**Keywords:** Amazonian bioeconomy, oleoresins, *Eperua oleifera* Ducke, *Copaifera multijuga* Hayne, Green Chemistry, DART-MS

## Abstract

Oleoresins are complex natural lipophilic matrices traditionally analyzed using chromatographic techniques that require extensive sample preparation, derivatization, and authentic standards. Amazonian oleoresins from *Copaifera* and *Eperua* species (Fabaceae) represent valuable bioresources with recognized pharmacological potential, largely attributed to diterpenoids such as copalic and hardwickiic acids, as well as bioactive sesquiterpenes, including the cannabinoid β-caryophyllene. In this study, we present a proof-of-concept application of Direct Analysis in Real Time coupled with High-Resolution Mass Spectrometry (DART-HRMS) as a rapid, direct, and environmentally friendly approach for chemical fingerprinting and semi-targeted screening of the two most important amazonian oleoresins from these two genera: *Eperua oleifera* and *Copaifera multijuga*. Analyses were performed using a Q Exactive Orbitrap coupled to a DART ion source under optimized conditions. Hardwickiic acid was used as a model compound for method optimization, with optimal performance achieved at 200 °C and 100 V, yielding stable signal intensities (CV < 10%) and high mass accuracy (<1 ppm). The method enabled reproducible detection of diterpenic acids in both oleoresins, allowing differentiation of their chemical profiles and assessment of short-term stability under ambient conditions. In addition to diterpenes, free fatty acids were also detected, expanding the compositional characterization of these matrices. Compound annotation was performed based on accurate mass measurements and literature comparison, corresponding to Level 5 confidence according to established metabolomics criteria. Although the absence of chromatographic separation limits isomer discrimination and absolute quantification, DART-HRMS provides a rapid and solvent-free strategy for chemical fingerprinting and preliminary characterization of oleoresins. This approach aligns with Green Chemistry principles and shows strong potential as a screening and triage tool for quality control, chemotaxonomic studies, and sustainable valorization of Amazonian natural products.

## 1. Introduction

Amazonian resins are natural plant-derived substances widely used by traditional forest communities for a variety of purposes, including medicinal applications, incense, adhesives, and repellents [1,2,3]. Among the native resin-producing species traditionally employed for these uses, *Protium heptaphyllum* (Aubl.) Marchand (breu-branco), *Eperua oleifera* Ducke, and *Copaifera multijuga* Hayne (Copaiba trees) are particularly noteworthy [4,5,6]. Low-impact extractive practices combined with the use of clean technologies have proven to be economically viable and sustainable, enabling the exploitation of these resources without compromising ecological balance. In this context, resins emerge as key elements of the Amazonian bioeconomy, as they integrate environmental conservation, cultural valorization, and technological development [7,8].

Copaiba trees are native to tropical regions of Latin America and West Africa, with species distributed throughout Mexico and northern Argentina [9]. These trees grow slowly, reaching heights of 5 to 40 m, and produce copaiba oleoresin, an exudate composed of sap containing resin acids and volatile compounds [10]. In Brazil, copaiba oil is of great social, economic, and medicinal importance [11,12]. From a medicinal perspective, it exhibits antimicrobial, healing, and anti-inflammatory properties, and it is also widely used in the perfume and cosmetic industries, as well as in varnishes and paints [9,13]. In a similar context, the genus *Eperua* Aublet is distributed throughout Central Amazonia, covering the northern region, including parts of Manaus, other areas of the state of Amazonas, southern Ecuador, the Guianas, and Venezuela [14]. The main product obtained from *Eperua* species is resin, which is used in traditional medicine for its healing properties, comparable to those reported for the genus *Copaifera* L. This resin has been described as exhibiting healing, antifungal, and antibacterial activities [15,16].

Hardwickiic and copalic acids are recognized as two of the most abundant diterpenoid acids in the oleoresins of *Eperua oleifera* Ducke and *Copaifera multijuga* Hayne, respectively. These compounds belong to the clerodane- and labdane-type diterpenoid families, which are characterized by tricyclic and bicyclic carbon skeletons, respectively, derived from the geranylgeranyl pyrophosphate terpene biosynthetic pathway. Hardwickiic acid exhibits a clerodane-type carboxylate structure with a conjugated diene system, which contributes to its chemical stability and characteristic fragmentation pattern in mass spectrometry. This compound has been associated with anti-inflammatory, antimicrobial, and cytotoxic activities, highlighting its pharmacological relevance. Copalic acid, a structurally related labdane diterpenoid, is considered a chemotaxonomic marker of *Copaifera* species and is typically the predominant constituent of *C. multijuga* oleoresin, often accompanied by minor diterpenoids such as kaurenoic and hardwickiic acids [12,13]. Together, these compounds play a central role in defining the chemical and biological profiles of Amazonian oleoresins [1,14].

The growing medicinal and economic interest in oleoresins from *Copaifera* and *Eperua* species underscores the need for rapid, sustainable analytical strategies to evaluate their chemical biomarkers directly at the extraction site. These Amazonian trees produce complex mixtures of other bioactive diterpenes together with sesquiterpenes, which are widely used in both traditional and modern medicine [16]. However, ensuring quality, traceability, and environmental sustainability remains a challenge, especially given the geographical distance between Amazonian extraction areas and major industrial or research centers.

Generally, analyzing natural oleoresins using traditional gas chromatography (GC) or liquid chromatography (LC) methods requires several preparatory steps, including pH adjustments, extraction, solvent manipulation, fractionation, concentration, and derivatization [13,17]. Large signal intensities of starting material may be needed to offset sample loss in extensive processing. Additionally, degradation, oxidation, and other artifacts can occur during sample preparation [18].

Advances in analytical speed and environmental compatibility can be achieved through direct, real-time analysis combined with mass spectrometry, particularly using Direct Analysis in Real Time mass spectrometry (DART-MS) [19]. DART-MS is an advanced technology that has significantly impacted many fields of chemical analysis [20]. The importance of DART-MS in quality control lies in its ability to accelerate and enhance the detection, concentration estimation, and compositional analysis of different samples [21]. This technique can directly ionize compounds without sample preparation or dilution, enabling rapid and reliable identification across various matrices [22]. In natural products research, DART-MS is used for chemotaxonomic studies of lipids, alkaloids, and saccharides. It involves using chemical profiles of biological markers, such as metabolites or surface molecules, as a chemical fingerprint [23], as well as metabolomic studies in the analysis of complex mixtures [24].

In this context, the DART-HRMS technique aligns strongly with the principles of Green Chemistry by minimizing solvent use, reducing waste generation, and eliminating time- and resource-intensive sample preparation steps. Its ambient ionization capability enables real-time chemical fingerprinting of oleoresins, supporting guided, responsible extraction practices that help prevent overharvesting and forest degradation [25,26]. This approach directly supports several United Nations Sustainable Development Goals (SDGs), including responsible consumption and production by promoting eco-efficient analytical practices and sustainable use of natural products; climate action through reducing chemical waste and the carbon footprint of analytical workflows; life on land by fostering biodiversity conservation and sustainable forest management; and good health and well-being by ensuring the safety and efficacy of medicinal natural products [26]. By promoting the rational use of natural resources, conserving biodiversity, and strengthening a sustainable Amazonian economy, this strategy effectively bridges scientific innovation with environmental preservation and socio-economic development [4,5].

Overall, applying DART-HRMS as a rapid, green, and field-deployable technique fosters an integrated approach that bridges scientific innovation, environmental preservation, and socio-economic sustainability in the Amazon region. This work presents a proof-of-concept for a new strategy that enables rapid, specific, and reproducible analysis of resin using DART-HRMS.

## 2. Results and Discussion

### 2.1. DART-HRMS Analysis

#### 2.1.1. Optimization of the DART-HRMS Method Performance

The DART-HRMS method was optimized for hardwickiic acid ionization efficiency. In particular, the temperature of the carrier gas influences the DART ionization process. The temperature of the ionization gas provides thermal energy for ionization and induces the thermal desorption of analytes from solid samples. Therefore, at very high temperatures, the ion of interest may degrade, while at very low temperatures, proper ionization may not be induced due to insufficient energy [27].

**Figure 1:**
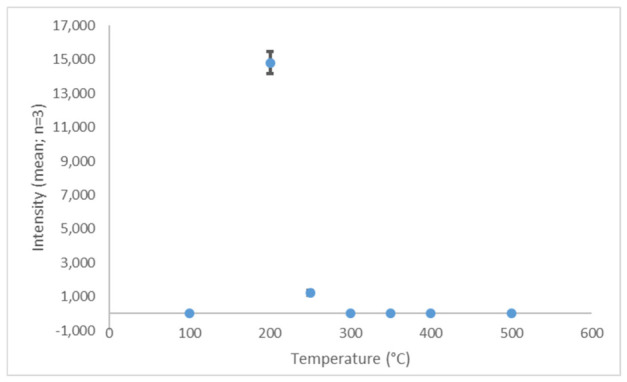
Evaluation of the ionizing gas temperature on the absolute intensity of *m*/*z* 315.1966 [M − H]^−^. Values represent the means of three technical replicates (*n* = 3).

DART ionization occurs in the gas phase following thermal desorption, which necessitates careful optimization of desorption conditions. The gas temperature was therefore optimized for hardwickiic acid, selected as the biomarker of *E. oleifera* oleoresin, as shown in Figure 1 [16]. The highest signal intensity was observed at 200 °C with precision (CV = 4.4%, *n* = 3). At low temperatures, thermal desorption may not reach a steady state, leading to an incorrect analyte concentration in the gas phase. Meanwhile, at high temperatures, desorption can occur too rapidly, causing signal loss as the vapor-phase analyte dissipates too quickly into the surroundings [22]. Therefore, selecting an optimal desorption temperature is critical to balance analyte release and signal stability, ensuring reliable detection and reproducibility in DART-HRMS analyses. The data are shown in Figure 1, and the raw data used to generate this graph are provided in Table S4 of the Supplementary Material.

**Figure 2:**
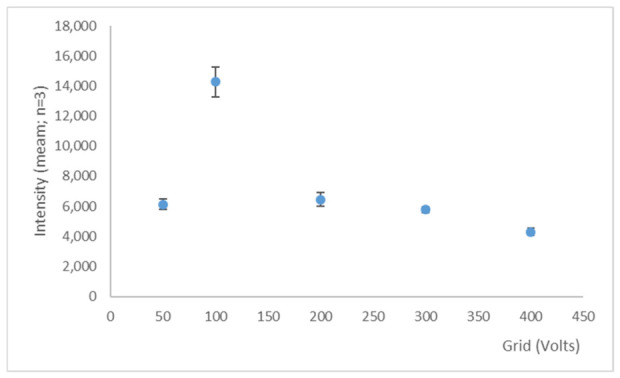
Evaluation of the DART grid voltage on the absolute intensity of *m*/*z* 315.1966 [M − H]^−^. Values represent the means of three technical replicates (*n* = 3).

Another critical parameter affecting ionization performance in DART is the grid voltage. The DART grid voltage is vital for preventing ion recombination, facilitating ion flow from the sample to the mass spectrometer, and providing a source of electrons via surface Penning ionization [27]. After establishing 200 °C as the optimal gas temperature, the grid voltage was tested within the range of 50 V to 400 V. The pseudomolecular ion intensity increased at 100 V (CV = 7.0%, *n* = 3). Beyond 200 V, a decline in signal intensity was observed without any increase in the relative standard deviation. Coefficient of variation values below 10% indicate that, despite a significant decrease in the signal intensity of the deprotonated ion, the ionization process for fresh resin remains consistent and accurate. The highest CV% value recorded was 7.1% at a GRID voltage of 200 V, and the lowest was 3.3% at 300 V. The data are shown in Figure 2, and the raw data used to generate this graph are provided in Table S5 of the Supplementary Material.

According to those results, the most effective instrumental conditions observed for hardwickiic acid analysis were a grid voltage of 100 V and a carrier gas temperature of 200 °C. All mass error values obtained in this experiment were below 1 ppm.

Taken together, these findings show that carefully optimizing both carrier gas temperature and grid voltage is crucial for maximizing ionization efficiency, accuracy, and consistency in DART-HRMS analysis. The combination of 200 °C and 100 V provided the optimal conditions, yielding stable signal intensities with low variability and high mass accuracy (<1 ppm) for hardwickiic acid. These optimized settings provide a reliable framework for analyzing diterpenic acids in complex oleoresin matrices.

#### 2.1.2. Analysis of Hardwickiic Acid in Oil Resin

Based on the exact mass determined by comprehensive acquisition and the mass spectra acquired by Data-Dependent Analysis (DDA), hardwickiic acid can be rapidly detected without the need for specific sample preparation. The sample’s homogeneous nature further facilitates direct analysis.

These variations reflect the inherent differences in ion formation processes: while electrospray ionization (ESI) promotes the generation of stable [M − H]^−^ ions through solution-phase deprotonation, DART produces ions via direct gas-phase reactions, resulting in lower pseudomolecular ion intensity and altered fragment distributions. In recent work [16], during the characterization of the chemical constituents of *E. oleifera* oleoresin using UHPLC-HRMS, hardwickiic acid was identified by ESI negative mode. The precursor ion *m*/*z* 315.1966 was fragmented in *m*/*z* 301.1820 (14%), *m*/*z* 285.18582 (11%), *m*/*z* 273.2223 (26%), and *m*/*z* 257.1909 (16%). The fragmentation profile of hardwickiic acid observed under UHPLC-HRMS conditions serves as a benchmark for comparison with DART-HRMS, where differences in ionization mechanisms between ESI and DART not only affect the intensity of pseudomolecular ions but also shape the diversity of fragment ions detected. In DART-MS, the pseudomolecular ion is much less intense. The fragmentation profile acquired with a normalized collision energy (NCE) of 30 is represented by the ions *m*/*z* 301.1820 (5%), *m*/*z* 285.1858 (20%), *m*/*z* 273.2223 (38%), and *m*/*z* 257.1909 (10%).

Electrospray ionization (ESI) and direct analysis in real time (DART) differ significantly in their ionization mechanisms, which influence ionization efficiency and fragmentation behavior. ESI is a soft ionization method that primarily produces intact pseudomolecular ions, preserving molecular integrity, making it ideal for polar and thermally labile compounds [27]. In contrast, DART employs a plasma-based process that uses Penning ionization, in which metastable species ionize analytes in open air. This mechanism provides broader ionization coverage and higher ionization efficiency but often results in greater in-source fragmentation [22,28]. While this may complicate direct detection, it also offers additional structural information, making DART more suitable for rapid, exploratory analyses. In contrast, ESI excels in preserving molecular ions for accurate mass determination [29].

#### 2.1.3. Detectability Assessment in Methanolic Extracts of *E. oleifera*

The detectability assessment in methanolic extracts of *E. oleifera* of hardwickiic acid under the optimized conditions was determined by analyzing the diluted extract in Full MS mode over three consecutive runs at a final concentration of 10 µg/mL. The presence of hardwickiic acid was confirmed through its pseudomolecular ion. The coefficient of variation across the three measurements was 6.1%, and the mass error remained below 1 ppm. Therefore, absolute intensity values on the order of 10^3^ are considered sufficiently robust to support stability-related inferences.

#### 2.1.4. Five-Day Stability Assessment of *E. oleifera* and *C. multijuga* on Quick Strip

The analysis across consecutive days demonstrated the resin’s stability even under environmental conditions. During the experimental period, the ambient temperature and relative humidity were 20.7 °C and 67.1%, respectively. In a previous study, we confirmed the absence of sesquiterpenes and monoterpenes in the resin of *E. oleifera* [16]. The lack of these volatile components likely contributes to the enhanced stability of the resin, as monoterpenes and sesquiterpenes are prone to evaporation and oxidation. As shown in Table 1, no significant differences in the absolute intensities of the target analyte were observed across consecutive days, yielding a coefficient of variation of 3.2%.

To support this conclusion, the same experiment was conducted with *C. multijuga* oleoresin, which contains sesquiterpenes and monoterpenes [16]. The data are presented in Table 2.

It is well known that hardwickiic acid is not the most abundant biomarker in copaiba oleoresin; however, the absolute intensity of its characteristic deprotonated ion fragment was detected in the last experiment (11 April 2025) at about twice the estimated limit of detection. According to Table 2, the 48% coefficient of variation supports our hypothesis that the presence of sesquiterpenes and monoterpenes affects oleoresin stability, leading to lower stability under environmental conditions compared to *E. oleifera* oleoresin.

Hardwickiic acid was selected as a model compound for method optimization and signal stability assessment due to its availability and well-characterized behavior under HRMS conditions.

To provide a more representative perspective for *C. multijuga*, copalic acid, recognized as a dominant diterpenoid marker of this species, was also screened under the same DART-HRMS conditions based on its exact mass. A consistent signal corresponding to its deprotonated ion was observed across the analyses; however, due to the absence of chromatographic separation, reference standards, and MS/MS confirmation for this compound, this observation is reported as a tentative annotation (Level 5 confidence).

The signal behavior of copalic acid (Table 3 and Table 4) followed a similar qualitative trend to that observed for hardwickiic acid, supporting the general stability pattern described for both oleoresins. In *E. oleifera* oleoresin, the copalic acid signal remained highly stable over the five-day period (CV = 1.8%), whereas a progressive decrease in signal intensity was observed for *C. multijuga* oleoresin (CV = 53%).

These differences may reflect intrinsic variations in matrix composition, particularly the presence of volatile mono- and sesquiterpenes in copaiba oleoresin, which may favor oxidative and evaporative processes under environmental conditions. In contrast, the chemically less volatile matrix of *E. oleifera* appears to provide greater stability for diterpenic constituents such as copalic acid.

An additional explanation may involve oxidative and resinification processes occurring in *C. multijuga* oleoresin during environmental exposure. Oleoresins rich in unsaturated mono- and sesquiterpenes are known to undergo oxidation and polymerization reactions, potentially altering analyte desorption efficiency and ionization behavior under DART conditions. Such processes may contribute to the progressive decrease in copalic acid signal intensity observed over consecutive days.

Nevertheless, these observations remain qualitative, and no quantitative or compound-specific comparative conclusions are drawn.

#### 2.1.5. Assessment of Hardwickiic Acid Detectability in Repeated Resin Analyses

Hardwickiic acid was detectable in the *E. oleifera* sample for up to four days of reanalysis, as shown in Table 5.

A decrease in signal intensity was observed as expected due to sample desorption; however, detection remained above the method’s limit of detection even on the fifth consecutive day. The increase in mass error was observed throughout the experiment, consistent with the Orbitrap analysis. In this mass spectrometer, mass accuracy (ppm) depends on the analyte: lower analyte concentrations generally lead to higher mass errors due to lower signal-to-noise ratios, whereas higher concentrations improve mass accuracy [30].

This experiment was repeated with *C. multijuga*. However, on the second day of reanalysis, the characteristic fragment of the deprotonated pseudomolecular ion of hardwickiic acid could no longer be detected, as it fell below the method’s established limit of detection.

#### 2.1.6. Detection of Acidic Diterpenes, Their Corresponding Methyl Esters, and Other Compounds in *E. oleifera* and *C. multijuga* by DART-HRMS

To our knowledge, this is the first report to use DART-HRMS for the chemical fingerprinting of an oleoresin. In this study, we took a targeted approach to analyze the chemical composition of *E. oleifera* and *C. multijuga*, relying on accurate mass searches of deprotonated and protonated pseudomolecular ions previously reported in the literature [16]. The high-resolution capability of DART-HRMS coupling enabled the direct detection of these ions in the crude matrix, eliminating the need for prior chromatographic separation. According to the confidence level framework proposed by Schymanski et al. (2014) [31], targeted analyses without chromatographic separation typically achieve Level 5 confidence in compound detection. The putative annotation is based on exact mass accuracy and structural identification, supported by literature matches.

**Figure 3:**
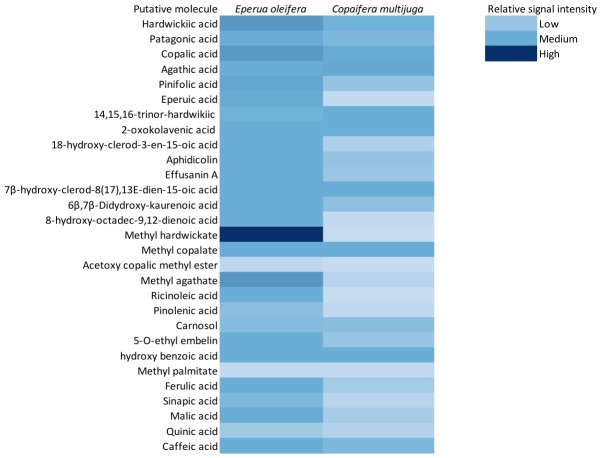
Heatmap showing the relative signal intensity of tentatively annotated diterpenic acids, their corresponding methyl esters, and related compounds detected by DART-HRMS in Eperua oleifera and Copaifera multijuga. The color scale represents relative signal intensity under identical experimental conditions (low to high). Values are qualitative and should not be interpreted as an absolute or quantitative composition.

These findings demonstrate the potential of DART-HRMS as a fast and reliable tool for oleoresin analysis, broadening its applications beyond previous studies in natural product research, including propolis and other resinous matrices [32]. The detailed results of this approach are summarized in Figure 3.

The DART-HRMS method presented here is not designed to distinguish between isomers (e.g., patagonic acid and 14-deoxy-11,12-didehydro-andrographolide, 16-oxo-13,14H-hardwickiic acid, or copalic acid and kovalenic acid), nor any other isomers present in the matrix. However, this limitation is compensated for by the technique’s ability to detect molecules without requiring sample preparation. Moreover, this triage approach can be highly advantageous when implemented before the application of time-consuming confirmatory analyses. Table S1 in the Supplementary Materials lists possible isomeric assignments, along with individual signal intensities and mass errors for each analyte in both samples.

In Table S3 (Supplementary Material), the tentative annotation of diterpenoids, fatty acids, phenolic acids, and related oxygenated metabolites was supported by characteristic fragmentation pathways observed under DART-HRMS/MS in negative ion mode. In general, diterpenic acids exhibited deprotonated ions [M − H]^−^, followed by fragmentation dominated by dehydration (−18 Da), decarboxylation (−44 Da), and subsequent cleavage of the diterpenoid skeleton. Hydroxylated diterpenes, including kaurane- and clerodane-type structures, frequently showed sequential water losses due to hydroxyl substituents, whereas diterpenic acids with free carboxylic groups showed the characteristic neutral loss of CO_2_. Additional lower-intensity ions were associated with rearrangements and fragmentation of the polycyclic diterpenoid framework.

Methyl ester derivatives, including methyl hardwickate, methyl copalate, and methyl agathate, showed fragmentation patterns consistent with methanol loss (−32 Da), ester rearrangements, and cleavage of the diterpenoid skeleton. Similarly, acetylated diterpenes such as acetoxy copalic methyl ester produced characteristic fragment ions corresponding to the neutral loss of acetic acid (−60 Da), supporting the presence of acetoxy substituents in the molecular structure.

Hydroxylated and unsaturated fatty acids predominantly underwent fragmentation via dehydration and decarboxylation, followed by cleavage along the aliphatic chain. In hydroxylated compounds, water loss was a dominant fragmentation pathway, whereas polyunsaturated fatty acids produced additional low-intensity fragments associated with allylic and vinylic cleavages.

Phenolic and hydroxycinnamic acids, including caffeic, ferulic, sinapic, and p-hydroxybenzoic acids, showed characteristic fragmentation patterns dominated by neutral loss of CO_2_ from the carboxylic group. Methoxylated derivatives, such as ferulic and sinapic acids, also produced fragment ions corresponding to methyl radical loss (−15 Da), consistent with cleavage of methoxy substituents on the aromatic ring.

Organic acids such as malic and quinic acids mainly exhibited fragmentation pathways involving dehydration and decarboxylation reactions, which are typical for polyhydroxylated carboxylic acids under collision-induced dissociation conditions.

Overall, the observed fragmentation behavior was chemically consistent with the proposed molecular classes and supported the tentative annotation of the detected compounds. However, because chromatographic separation and authentic reference standards were absent, all compound assignments should be regarded as tentative and reported at Level 5 confidence.

#### 2.1.7. Detection of Free Fatty Acids in *E. oleifera* and *C. multijuga* by DART-HRMS

**Figure 4:**
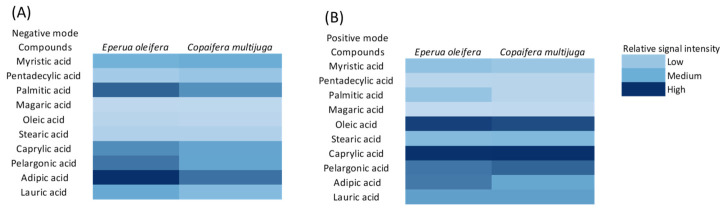
Heatmap showing the relative signal intensity of tentatively annotated free fatty acids detected by DART-HRMS in *Eperua oleifera* and *Copaifera multijuga*. (**A**) Negative ion mode and (**B**) positive ion mode. The color scale represents relative signal intensity under identical experimental conditions (low to high). Values are qualitative and are intended for comparative purposes only.

In addition to the major diterpenic constituents, free fatty acids were investigated in both resins using DART-HRMS. This analysis aimed to expand the chemical characterization of the resin, as minor lipid components may contribute to its physicochemical properties or originate from associated endophytic microorganisms, providing a more comprehensive metabolomic profile. The obtained results are presented in Figure 4A (negative mode) and 4B (positive mode). The data on the fatty acids present in *Eperua oleifera* are presented in Table S2 of the Supplementary Materials.

The detection of free fatty acids in oleoresins can be rationalized by multiple, non-mutually exclusive mechanisms. First, plants naturally produce a wide variety of lipids, including structural phospholipids, glycolipids, cuticular waxes, and storage triacylglycerols, all of which are ultimately derived from fatty acids [33,34]. Although diterpenes and their corresponding methyl esters have been described as the dominant constituents of oleoresins, minor lipid fractions may also be present and can contribute to physicochemical properties such as viscosity and protection against desiccation [35].

Second, the role of associated microbiota cannot be excluded. Endophytic bacteria and fungi inhabiting Fabaceae species are known to biosynthesize fatty acid derivatives, which may contribute to the observed chemical profile [36,37]. Finally, analytical artifacts should also be considered. In DART- or ESI-based mass spectrometry, in situ transesterification of free fatty acids may occur in the presence of trace signal intensities of methanol, leading to the formation of fatty acid methyl esters (FAMEs) during ionization. However, this possibility is minimized here, as the direct analysis of the resin was performed without methanol dilution [38].

DART-HRMS enabled the detection of free fatty acids in both positive and negative ion modes [39]. However, it is important to emphasize that signal intensity in DART-HRMS does not directly reflect compound concentration or absolute signal intensity, due to the lack of chromatographic separation, ionization efficiency differences, and absence of calibration with authentic standards. Therefore, the observed signal intensities are interpreted here only in a qualitative or semi-comparative manner within the same experimental conditions.

The detected differences in signal patterns between *E. oleifera* and *C. multijuga* suggest distinct chemical fingerprints; however, these observations should not be interpreted as quantitative compositional differences. Similarly, although variations in free fatty acid signals may be associated with differences in matrix composition or sample history, no direct conclusions regarding oxidative processes, aging, or stability can be drawn without targeted analytical validation.

Within a metabolomics framework, the detected free fatty acids are therefore reported as tentatively annotated compounds (Level 5 confidence), based solely on accurate mass measurements in both ionization modes and literature comparison [32].

## 3. Materials and Methods

### 3.1. Plant Material

The oil resin from *Eperua oleifera* was collected in Manicoré, in September/2023, and *Copaiba multijuga* was collected in Manaus, in September/2023, both in Amazonas State, Brazil. The research project was registered on the National System for Governance of Genetic Heritage and Associated Traditional Knowledge (SisGen AAC8B84/Voucher number 230699 and AAB3AA1/Voucher number INPA82418). Hardwickic acid was previously isolated and its structure elucidation at our research group.

### 3.2. Sample Preparation for DART-HRMS

#### 3.2.1. Direct Sample Analysis

The semi-solid sample of each oil resin was pipetted onto the QuickStrip^TM^ sample card (Bruker Corporation, Billerica, MA, USA) and dried at room temperature. The QuickStrip^TM^ will then be placed in the sample holder of the DART source. Methanol (GC grade) obtained from Tedia (Fairfield, OH, USA) was used, as the same approach was applied to the negative controls (methanol). Each sample replicate was intercalated with a negative control on the QuickStrip^TM^. Each experimental point was analyzed in triplicate (*n* = 3).

#### 3.2.2. Dilute Sample Analysis

To prepare the oleoresin extract, 1 mg of crude resin was dissolved in methanol and subsequently diluted 100-fold to obtain a final concentration of 10 µg/mL.

For each species (*Eperua oleifera* and *Copaifera multijuga*), a single oleoresin batch was analyzed. The samples were obtained from previously characterized materials [16].

All measurements were performed as technical replicates under identical experimental conditions using the DART-HRMS system. Replicate analyses were conducted on consecutive days to evaluate signal repeatability and short-term stability.

No biological replicates (i.e., independent samples collected from different individuals or locations) were included in this proof-of-concept study. Therefore, the results reflect analytical repeatability and matrix-specific signal behavior rather than biological variability.

### 3.3. DART-HRMS Method Performance Evaluation

Method performance was evaluated for hardwickiic acid based on the precision obtained from the analysis of three consecutive samples. The semi-solid sample was placed in front of the DART-MS holder. The media, standard deviation, and coefficient of variation were calculated for each triplicate.

#### 3.3.1. Assessment of the Limit of Detection in Methanolic Extracts of *E. oleifera*

To evaluate the limit of detection of hardwickiic acid under the optimized conditions, 1 mg of crude resin was diluted in methanol and subsequently diluted 100-fold to obtain an extract with a final concentration of 10 µg/mL. The oil resin extract was pipetted onto the QuickStrip^TM^ card and dried at room temperature. The Quick-Strip^TM^ will then be placed in the sample holder of the DART source. Three consecutive analyses of this extract were performed in Full MS mode. The media, standard deviation, and coefficient of variation were calculated for each triplicate.

#### 3.3.2. Five-Day Stability Assessment of *E. oleifera* and *C. multijuga* on Quick Strip

To evaluate the stability, 5 µL of each oleoresin was deposited at five consecutive positions on the QuickStrip, with an empty position between each sample. Analyses were conducted over five successive mornings under controlled temperature and humidity conditions. Each well was analyzed on each of the five days.

#### 3.3.3. Assessment of Hardwickiic Acid Detectability in Repeated Resin Analyses

The oil resin was applied to a single position on the QuickStrip and analyzed on consecutive days without reapplication. In this approach, we assessed how many analyses of the same well containing the resin could still detect Hardwickiic acid.

### 3.4. DART-HRMS Analysis and Instrument Conditions

High-resolution mass spectra QExactive Orbitrap mass spectrometer (Thermo Fisher Scientific, Bremen, Germany) coupled with an IonSense^®^ DART^®^ JumpShot source (Saugus, MA, USA) was used. Mass spectra were acquired in negative and positive ionization modes using Helium (He) as the carrier and ionization gas. The distance between the MS inlet and the DART gunshot was 3 cm. The DART ion source was set as follows: needle potential, 300 V; grid potential, 100 V; autosampler velocity, 1 mm/s; and the ionization gas temperature was evaluated at 100 °C, 200 °C, 250 °C, 300 °C, 350 °C, 400 °C, and 500 °C. The DART instrumental setup for standard ionization was initially assessed using a direct analysis of resin, a signal intensity equivalent to 10 μL. The mass spectrometer, operating in negative and positive modes, was calibrated daily with a manufacturer’s calibration solution (Thermo Fisher Scientific, Bremen, Germany).

Full-scan data were acquired in a range of *m*/*z* 70–*m*/*z* 1050 at a resolution of 70,000 full widths at half maximum (FWHM), automatic gain control (AGC) of 1 × 10^6^, and maximum injection time (IT) of 100 ms.

#### Data Processing Analysis

Data were acquired and processed using Thermo Scientific XCalibur 3.2 software (Thermo Fisher Scientific, Austin, TX, USA), with a mass tolerance of ±5 ppm.

## 4. Conclusions

The present study demonstrated the potential of DART-HRMS as a rapid, efficient, and environmentally friendly approach, aligned with the principles of Green Chemistry, for the chemical profiling of Amazonian oleoresins from *Eperua oleifera* and *Copaifera multijuga*. The possibility of direct analysis of complex matrices, with minimal or no sample preparation, significantly reduced analysis time, solvent consumption, and the risk of analyte degradation, highlighting the applicability of the technique for rapid and high-throughput analyses.

The use of hardwickiic acid as a model compound demonstrated high signal reproducibility and excellent mass accuracy under optimized analytical conditions. In addition, the detection of diterpenic acids, sesquiterpenes, free fatty acids, and other constituents enabled the establishment of characteristic chemical fingerprints for both oleoresins, contributing to the identification of relevant biomarkers and the chemical differentiation between the evaluated species. The inclusion of copalic acid as a species-specific marker for *C. multijuga* provided further support for the qualitative interpretation of the observed chemical profiles.

Although the technique presents limitations related to the absence of chromatographic separation, the discrimination of structural isomers, and definitive structural confirmation of compounds, DART-HRMS demonstrated high sensitivity and reliability in the detection of characteristic molecular ions. Compound annotations were based on accurate mass measurements, MS/MS fragmentation, and comparison with literature data, and were therefore considered putative identifications. Furthermore, due to differences in ionization efficiency and the absence of calibration with authentic standards, the results were interpreted only qualitatively or semi-quantitatively.

In this context, DART-HRMS stands out as a powerful initial screening tool for exploratory studies, quality control, natural product authentication, and chemotaxonomic investigations, particularly for in situ and large-scale analyses. Nevertheless, complementary techniques such as LC-MS and GC-MS coupled with chromatographic separation remain necessary for structural confirmation, isomer discrimination, and more robust quantitative analyses. Therefore, the proposed approach represents a promising, cost-effective, and environmentally sustainable analytical alternative for the valorization and traceability of Amazonian oleoresins.

## Figures and Tables

**Table 1:** Five-day stability assessment of hardwickiic acid in *E. oleifera* on Quickstrip.

Experiment Dates	[M − H]^−^	Mass Error (ppm)	Absolute Intensity	SD	Mean	CV%
7 April 2025	315.1967	0.32	16,280	518	16,168	3.2
8 April 2025	315.1968	0.48	16,910			
9 April 2025	315.1967	0.25	15,980			
10 April 2025	315.1967	0.32	16,190			
11 April 2025	315.1968	0.63	15,480			

**Table 2:** Five-day stability assessment of hardwickiic acid in *C. multijuga* on QuickStrip.

Experiment Dates	[M − H]^−^	Mass Error (ppm)	Absolute Intensity	SD	Mean	CV%
7 April 2025	315.1969	0.95	8354	2497	5196	48
8 April 2025	315.1972	1.90	6910			
9 April 2025	315.1970	1.24	5209			
10 April 2025	315.1980	4.44	2967			
11 April 2025	315.1971	1.59	2540			

**Table 3:** Five-day stability assessment of copalic acid in *E. oleifera* oleoresin on QuickStrip.

Experiment Dates	[M − H]^−^	Mass Error (ppm)	Absolute Intensity	SD	Mean	CV%
7 April 2025	303.2330	0.33	61,309	1129	61,272	1.8
8 April 2025	303.2327	−0.66	59,876			
9 April 2025	303.2329	0.00	60,823			
10 April 2025	303.2328	−0.33	62,987			
11 April 2025	303.2327	−0.66	61,365			

**Table 4:** Five-day stability assessment of copalic acid in *C. multijuga* oleoresin on QuickStrip.

Experiment Dates	[M − H]^−^	Mass Error (ppm)	Absolute Intensity	SD	Mean	CV%
7 April 2025	303.2328	−0.33	58,972	18,650	35,182	53
8 April 2025	303.2325	−1.32	49,834			
9 April 2025	303.2327	−0.66	30,890			
10 April 2025	303.2330	0.33	20,236			
11 April 2025	303.2326	−0.99	15,978			

**Table 5:** Assessment of hardwickiic acid detectability in repeated *E. oleifera* analyses.

Experiment Dates	[M − H]^−^	Mass Error (ppm)	Absolute Intensity
7 April 2025	315.1967	0.32	16,280
8 April 2025	315.1968	0.63	4676
9 April 2025	315.1960	−1.90	3428
10 April 2025	315.1959	−2.22	3205
11 April 2025	315.1952	−4.44	2098

## Data Availability

The original contributions presented in this study are included in the article. Further inquiries can be directed at the corresponding author.

## Supplementary Materials

The following supporting information can be downloaded at https://www.mdpi.com/article/10.3390/plants15111651/s1. Table S1: Metabolic fingerprint of *Eperua oleifera* and *Copaifera multijuga* obtained using DART-HRMS. Table S2: Free fatty acids assigned in *Eperua oleifera* and *Copaifera multijuga*, obtained using DART-HRMS. Table S3: Compounds fragmentations of *Eperua oleifera* and *Copaifera multijuga* obtained using DART-HRMS in negative mode. Table S4. Raw intensity values, mean intensity, standard deviation, and coefficient of variation obtained for hardwickiic acid during DART source temperature optimization. Table S5. Raw intensity values, mean intensity, standard deviation, and coefficient of variation obtained for hardwickiic acid during DART grid voltage optimization.
