# Supplementary material for: Medicinal Amazonian Oleoresins: An Eco-Friendly Chemical Fingerprinting Method
Source: Plants (Basel). 2026 May 28;15(11):1651. doi: 10.3390/plants15111651 (PMC13259250; doi:10.3390/plants15111651)
Supplement: Supplementary file 1 [file plants-15-01651-s001.zip › plants-4301695-supplementary.pdf]

Supplementary Material

Medicinal Amazonian Oleoresins: An Eco-Friendly Chemical Fingerprinting Method

Rayssa Ribeiro 1, Gabriel Reis Alves Carneiro 2, Henrique Marcelo Gualberto Pereira 2, Monica Costa Padilha 2 and Valdir F. Veiga-Junior 1,*

1 Military Institute of Engineering, Rio de Janeiro , Praça General Tibúrcio, 80, Praia Vermelha, Urca, Rio de Janeiro 22290-270, RJ, Brazil

2 Brazilian Doping Control Laboratory (LBCD/IQ—UFRJ), Chemistry Institute, Federal University of Rio de Janeiro, Rio de Janeiro 21941-909, RJ, Brazil

----

*Corresponding author:

Full Prof. Dr. Valdir Veiga Junior

Email: valdir.veiga@gmail.com

Tel.: + 55 (21) 99115-8799

List of Supplementary Material

- **Table S1: Metabolic fingerprint of *Eperua oleifera* and *Copaifera multijuga* obtained using DART-HRMS.**

Putative Molecule	Molecule Formula [M]	Mode	Precursor ion ([M-H]⁻ /Mass error (ppm)		Absolute intensity/oleoresin		Chemical Class
			Eperua oleifera	*Copaifera multijuga*	*Eperua oleifera*	*Copaifera multijuga*	
Hardwickiic acid	$C_{20}H_{28}O_3$	(-)	315.1966 / (0)	315.1958 / (-2.5)	1962725	10643	Diterpene
Patagonic acid / 14-Deoxy-11,12-didehydro-andrographolide / 16-oxo-13,14H-hardwikiic acid	$C_{20}H_{28}O_4$	(-)	331.1912 / (-0.9)	331.1909 / (-1.8)	76683	8956	Diterpene
Copalic / Kovalenic / (-)-clerod-7,13E-diene-15-oic acid	$C_{20}H_{32}O_2$	(-)	303.2326 / (-1.0)	303.2323 / (2.0)	1872957	267135	Diterpene
Agathic / 12-hydroxy-7-carboxy-abiet-8(13)-en-18-oic acid	$C_{20}H_{30}O_4$	(-)	333.2070 / (-0.3)	333.2063 / (-2.4)	184585	539173	Diterpene
Pinifolic / Clerod-3-en-15,18-dioic acid / Ent-16β,17-dihydroxy-19-kaurenoic acid	$C_{20}H_{32}O_4$	(-)	335.2224 / (-1.2)	335.2224 / (-1.2)	613006	6221	Diterpene
Eperuic / Cativic acid	$C_{20}H_{34}O_2$	(-)	305.2482 / (-1.3)	305.2476 / (-3.3)	259719	512	Diterpene
14,15,16-trinor-hardwikiic / nor-hardwickiic acid / Embelin	$C_{17}H_{26}O_4$	(-)	293.1757 / (-0.3)	293.1754 / (-1.4)	10643	18937	Diterpene
2-oxokolavenic acid / 7-oxo-labda-8-en-15-oic acid	$C_{20}H_{30}O_3$	(-)	317.2121 / (-0.3)	317.2114 / (-2.5)	44626	70235	Diterpene
18-hydroxy-clerod-3-en-15-oic acid	$C_{20}H_{34}O_3$	(-)	321.2431 / (-1.2)	321.2438 / (0.9)	20297	3113	Diterpene
Aphidicolin	$C_{20}H_{34}O_4$	(-)	337.2374 / (-3.0)	337.2376 / (-2.4)	67360	6167	Diterpene

Effusanin A	$C_{20}H_{28}O_5$	(-)	347.1861 / (-0.9)	347.1856 / (-2.3)	166866	5646	Diterpene
(-)-7β-hydroxy-clerod-8(17),13E-dien-15-oic acid	$C_{20}H_{32}O_3$	(-)	319.2274 / (-1.6)	319.2276 / (-2.8)	45443	132207	Diterpene
6β,7β-Didydroxy-kaurenoic acid	$C_{20}H_{30}O_4$	(-)	333.2071 / (0)	333.2069 / (-0.6)	77980	7169	Diterpene
8-hydroxy-octadec-9,12-dienoic acid	$C_{18}H_{32}O_3$	(-)	295.2276 / (-1.0)	295.2267 / (-4.1)	14023	589	Diterpene
Methyl hardwickate	$C_{21}H_{30}O_3$	(-)	329.2122 / (0)	n.d.*	10807561	-	Diterpene
Methyl copalate	$C_{21}H_{34}O_2$	(-)	317.2486 / (0.3)	317.2482 / (-1.9)	187562	132207	Diterpene
Acetoxy copalic methyl ester	$C_{23}H_{36}O_4$	(-)	375.2541 / (1.9)	n.d.*	1124	-	Diterpene
Methyl agathate	$C_{21}H_{30}O_4$	(-)	347.2219 / (0.6)	347.2219 / (0.6)	2100506	2126	Diterpene
Ricinoleic acid	$C_{18}H_{34}O_3$	(-)	297.2434 / (-0.3)	n.d.*	34341	-	Unsaturated fatty acids
Pinolenic / a-Linolenic acid	$C_{18}H_{30}O_2$	(-)	277.2170 / (-1.1)	277.2169 / (-1.4)	7673	987	Polyunsaturated fatty acid
Carnosol	$C_{20}H_{26}O4$	(-)	329.1756 / (-0.6)	329.1758 / (0)	8177	7882	Diterpene
5-O-ethyl embelin	$C_{19}H_{30}O_4$	(-)	321.2069 / (-0.6)	321.2067 / (-1.2)	12278	5677	Quinone
ρ-hydroxy benzoic acid	$C_7H_6O_3$	(-)	137.0243 / (-0.7)	137.0243 / (0.7)	115534	23670	Phenolic acid
Methyl palmitate	$C_{17}H_{34}O_2$	(-)	269.2485 / (-0.4)	269.2481 / (-1.9)	667	653	Fatty acid esters
Ferulic acid	$C_{10}H_{10}O_4$	(-)	193.0497 / (-4.7)	193.0495 / (-3.6)	20931	4293	Phenolic acid
Sinapic acid	$C_{11}H_{12}O_5$	(-)	223.0606 / (-2.7)	223.0602/ (-4.5)	9113	1922	Phenolic acid

Malic acid	$C_4H_6O_5$	(-)	133.0141 / (0)	133.0142 / (0)	127718	3943	Phenolic acid
Quinic acid	$C_7H_{12}O_6$	(-)	191.0552 / (-4.7)	191.0559 / (-1.0)	4812	2587	Phenolic acid
Caffeic acid	$C_9H_8O_4$	(-)	179.0341 / (0.6)	179.0338 / (-1.1)	13787	9026	Phenolic acid

*n.d. = not detected

Table S2: Free fatty acids assigned in *Eperua oleifera* and *Copaifera multijuga*, using DART-HRMS.

Compound	Molecular Formula [M]	Precursor íon [M-H]$^-$ / Absolute intensity / Mass error (ppm)		Precursor ion [M-H]$^+$ / Absolute intensity / Mass error (ppm)	
		Eperua oleifera	*Copaifera multijuga*	*Eperua oleifera*	*Copaifera multijuga*
Myristic acid (C14:0)	$C_{14}H_{28}O_2$	227.2011 / 5741 / (-2.2)	227.2009 / 6603 / (-3.1)	229.2161 / 3370 / (-0.4)	229.2161 / 2806 / (-0.4)
Pentadecylic acid (C15:0)	$C_{15}H_{30}O_2$	241.2167 / 2441 / (-2.5)	241.2166 / 3152 / (-2.9)	243. 2319 / 872 / (0.4)	243.2317 / 982 / (-0.4)
Palmitic acid (C16:0)	$C_{16}H_{32}O_2$	255.2327 / 34281 / (-0.8)	255.2324 / 17081 / (-2.0)	257.2471 / 3131 / (-1.6)	257.2475 / 1009 / (0)
Magaric acid (C17:0)	$C_{17}H_{34}O_2$	269.2485 / 567 / (-0.4)	269.2481 / 653 / (-1.9)	271.2630 / 385 / (-0.7)	271.2626 / 410 / (-2.2)
Oleic acid (C18:1)	$C_{18}H_{34}O_2$	281.2481 / 999 / (-1.8)	281.2480 / 802 / (-2.1)	283.2631 / 25295 / (-0.4)	283.2631 / 23192 / (-0.4)
Stearic acid (C18:0)	$C_{18}H_{36}O_2$	283.2641 / 1509 / (-0.4)	283.2641 / 1530 / (-0.4)	285.2787 / 4236 / (-0.4)	285.2790 / 4480 / (0.7)
Caprylic acid (C8:0)	$C_8H_{16}O_2$	143.1072 / 18423 / (-3.5)	143.1075 / 9838 / (-1.4)	145.1223 / 27973 (0)	145.1223 / 28050 / (0)
Pelargonic acid (C9:0)	$C_9H_{18}O_2$	157.1232 / 27082 / (-1.3)	157.1232 / 9549 / (-1.3)	159.1379 / 15723 / (0)	159.1379 / 18982 / (0)
Adipic acid	$C_6H_{10}O_4$	145.0407 / 52269 / (-1.3)	145.0509 / 28185 / (2.1)	147.0652 / 15206 / (0)	147.0652 / 7074 / (0)
Lauric acid (C12:0)	$C_{12}H_{24}O_2$	199.1699 / 8299 / (-2.0)	199.1697 / 4609 / (-3.0)	201.1849 / 8540 / (0)	201.1848 / 8519 / (-0.5)

Table S3: Compounds fragmentations of *Eperua oleifera* and *Copaifera multijuga* obtained using DART-HRMS in negative mode.

Putative Molecule	Molecule Formula [M]	Precursor ion ([M-H]$^-$)		Fragment ions / Intensity (%)	
		Eperua oleifera	*Copaifera multijuga*	*Eperua oleifera*	*Copaifera multijuga*
Hardwickiic acid	$C_{20}H_{28}O_3$	315.1966	315.1958	301.1820 (5%)/ 285.1858 (20%)/ 273.2233 (38%)/ 257.1909 (10%)	301.1818 (4%)/ 285.1862 (18%)/ 273.2229 (35%)/ 257.1911 (8%)
Patagonic acid / 14-Deoxy-11,12-didehydro-andrographolide / 16-oxo-13,14H-hardwikiic acid	$C_{20}H_{28}O_4$	331.1912	331.1909	287.2017 (30%)/ 313.1809 (60%)/	287.2016 (25%)/ 313.1806 (50%)
Copalic / Kovalenic / (-)-clerod-7,13E-diene-15-oic acid	$C_{20}H_{32}O_2$	303.2326	303.2323	285.2224 (25%)/ 259.2432 (15%)/ 241.2326 (10%)	285.2222 (22%)/ 259.2430 (12%)/ 241.2322 (8%)
Agathic / 12-hydroxy-7-carboxy-abiet-8(13)-en-18-oic acid	$C_{20}H_{30}O_4$	333.2070	333.2063	315.1966 (60%)/ 289.2173 (28%)	315.1964 (60%)/ 289.2171 (25%)
Pinifolic / Clerod-3-en-15,18-dioic acid / Ent-16β,17-dihydroxy-19-kaurenoic acid	$C_{20}H_{32}O_4$	335.2224	335.2224	317.2122 (40%)/ 291.2330 (90%)/ 273.2224 (60%)	317.2121 (32%)/ 291.2329 (90%)/ 273.2222 (62%)
Eperuic / Cativic acid	$C_{20}H_{34}O_2$	305.2482	305.2476	287.2380 (60%)/ 261.2592 (40%)/ 243.2486 (5%)	287.2375 (60%)/ 261.2590 (35%)/ 243.2482 (6%)
14,15,16-trinor-hardwikiic / nor-hardwickiic acid / Embelin	$C_{17}H_{26}O_4$	293.1757	293.1754	275.1653 (40%)/ 249.1860 (25%)/ 331.1755 (10%)	275.1651 (28%)/ 249.1858 (20%)/ 331.1752 (8%)
2-oxokolavenic acid / 7-oxo-labda-8-en-15-oic acid	$C_{20}H_{30}O_3$	317.2121	317.2114	299.2017 (40%)/ 273.2224 (30%)/ 255.2118 (10%)	299.2015 (35%)/ 273.2222 (25%)/ 255.2116 (6%)

18-hydroxy-clerod-3-en-15-oic acid	$C_{20}H_{34}O_3$	321.2431	321.2438	303.2329 (60%)/ 277.2537 (30%)/ 259.2431 (10%)	303.2327 (55%)/ 277.2535 (28%)/ 259.2430 (8%)
Aphidicolin	$C_{20}H_{34}O_4$	337.2374	337.2376	319.2278 (70%)/ 301.2173 (30%)/ 283.2067 (20%)	319.2275 (62%)/ 301.2170 (29%)/ 283.2065 (18%)
Effusanin A	$C_{20}H_{28}O_5$	347.1861	347.1856	329.1758 (50%)/ 303.1966 (35%)/ 285.1860 (10%)	329.1756 (40%)/ 303.1963 (32%)/ 285.1862 (9%)
(-)-7β-hydroxy-clerod-8(17),13E-dien-15-oic acid	$C_{20}H_{32}O_3$	319.2274	319.2270	301.2173 (60%)/ 275.2380 (35%)/ 257.2274 (15%)	301.2170 (55%)/ 275.2384 (38%)/ 257.2279 (16%)
6β,7β-Dihydroxy-kaurenoic acid	$C_{20}H_{30}O_4$	333.2071	333.2069	315.1966 (70%)/ 297.1860 (35%)/ 289.2173 (25%)/ 271.2067 (10%)	315.1964 (68%)/ 297.1858 (33%)/ 289.2170 (25%)/ 271.2067 (10%)
8-hydroxy-octadec-9,12-dienoic acid	$C_{18}H_{32}O_3$	295.2276	295.2267	277.2173 (65%)/ 251.2380 (35%)/ 233.2274 (15%)	277.2170 (60%)/ 251.2376 (30%)/ 233.2272 (15%)
Methyl hardwickate	$C_{21}H_{30}O_3$	329.2122	n.d.*	297.1860 (60%)/ 285.1858 (35%)/ 273.2223 (20%)/ 257.1909 (10%)	n.d.*
Methyl copalate	$C_{21}H_{34}O_2$	317.2486	317.2482	285.2224 (50%)/ 273.2592 (30%)/ 257.2279 (10%)	285.2222 (45%)/ 273.2590 (26%)/ 257.2277 (10%)
Acetoxy copalic methyl ester	$C_{23}H_{36}O_4$	375.2541	n.d.*	315.2330 (70%)/ 343.2279 (35%)	n.d.*
Methyl agathate	$C_{21}H_{30}O_4$	347.2219	347.2219	315.1966 (65%)/ 303.1960 (50%)	315.1962 (60%)/ 303.1962 (45%)
Ricinoleic acid	$C_{18}H_{34}O_3$	297.2434	n.d.*	279.2329 (70%)/	n.d.*

				253.2537 (50%)/	
				235.2431 (15%)	
Pinolenic / a-Linolenic acid	$C_{18}H_{30}O_2$	277.2170	277.2169	233.2274 (60%)	233.2270 (55%)
Carnosol	$C_{20}H_{26}O4$	329.1756	329.1758	311.1653 (50%)/	311.1650 (45%)/
				285.1860 (30%)/	285.1858 (25%)/
				271.1704 (10%)	271.1702 (8%)
5-O-ethyl embelin	$C_{19}H_{30}O_4$	321.2069	321.2067	275.2016 (70%)/	275.2014 (65%)/
ρ-hydroxy benzoic acid	$C_7H_6O_3$	137.0243	137.0243	93.0346 (80%)	93.0342 (80%)
Methyl palmitate	$C_{17}H_{34}O_2$	269.2485	269.2481	237.2224 (40%)/	237.2227 (42%)/
				225.2592 (35%)	225.2589 (33%)
Ferulic acid	$C_{10}H_{10}O_4$	193.0497	193.0495	178.0271 (60%)	178.0269 (55%)
				149.0608 (40%)	149.0606 (35%)
				134.0373 (20%)	134.0370 (15%)
Sinapic acid	$C_{11}H_{12}O_5$	223.0606	223.0602	208.0377 (60%)/	208.0375 (55%)/
				179.0714 (75%)	179.0712 (78%)
				164.0479 (55%)	164.0473 (53%)
				149.0244 (10%)	149.0241 (8%)
Malic acid	$C_4H_6O_5$	133.0141	133.0142	115.0036 (60%)	115.0032 (55%)
				89.0244 (90%)	89.0242 (86%)
Quinic acid	$C_7H_{12}O_6$	191.0552	191.0559	173.0455 (60%)/	173.0453 (58%)/
				127.0399 (80%)/	127.0396 (76%)/
				111.0450 (20%)/	111.0448 (18%)/
				93.0344 (10%)	93.0342 (8%)
Caffeic acid	$C_9H_8O_4$	179.0341	179.0338	135.0452 (90%)	135.0450 (88%)
				107.0503 (60%)/	107.0500 (55%)/
				89.0244 (35%)	89.0241 (30%)

Table S4. Raw intensity values, mean intensity, standard deviation, and coefficient of variation obtained for hardwickiic acid during DART source temperature optimization.

Temperature (°C)	Intensity	SD	Mean	CV%
100	0<br>0<br>0	0	0	N/A*
200	15060<br>15320<br>14090	648	14823	4.4
250	1090<br>1370<br>1200	141	1220	11.6
300	0<br>0<br>0	0	0	N/A*
350	0<br>0<br>0	0	0	N/A*
400	0<br>0<br>0	0	0	N/A*
500	0<br>0<br>0	0	0	N/A*

*not applicable

SD: standard deviation; CV: coefficient of variation. *CV was not calculated (N/A) when the mean intensity was zero

Table S5. Raw intensity values, mean intensity, standard deviation, and coefficient of variation obtained for hardwickiic acid during DART grid voltage optimization.

GRID	Intensity	SD	Mean	CV%
50	6137	340	6137	5.5
100	14287	996	14287	7.0
200	6450	460	6450	7.1
300	5787	190	5787	3.3
400	4290	246	4290	5.7

SD: standard deviation; CV: coefficient of variation. *CV was not calculated when the mean intensity was zero.
